# Characterization of the complete mitochondrial genome of *Mucor indicus* Lendn. 1930 (Mucorales: Mucoraceae), isolated from the wine fermentation system

**DOI:** 10.1080/23802359.2024.2371376

**Published:** 2024-06-25

**Authors:** Yue Deng, Guangjiu Chen, Xuedong Bao, Jie He, Qiang Li

**Affiliations:** a Luzhou Vocational and Technical College, Luzhou, P. R. China; b School of Food and Biological Engineering, Chengdu University, Chengdu, P. R. China

**Keywords:** Mitochondrial genome, Evolution, Systemic development, Food, Fungi

## Abstract

*Mucor indicus* Lendn. 1930 has been widely used in food fermentation; however, its mitochondrial genome characteristics are not well understood. In this study, the complete mitochondrial genome of *M. indicus* was obtained, which was 61,400 bp in length with a GC content of 33%. The *M. indicus* mitochondrial genome was found to contain 14 core protein-coding genes, four free-standing ORFs, 18 intronic ORFs, 26 tRNAs, and two rRNA genes. Phylogenetic trees were generated for 25 early-differentiated fungi using the Bayesian inference (BI) method, which demonstrated that *M. indicus* is closely related to *Mucor piriformis*. This study provides useful information for the classification and evolution of *Mucor* species or other early-differentiated fungi.

## 1. Introduction

*Mucor indicus* Lendn. 1930, a species of the Mucoraceae family, is a common saprophyte fungus that is widely distributed in nature and includes soil, feces, hay, and air (Karimi and Zamani 2013). This fungus reproduces through sporangiospores and zygospores and has strong proteolytic activity. *M. indicus* has a wide range of applications in food fermentation (Xiang et al. 2019; Asachi et al. 2011). It can be used as a starter culture to hydrolyze proteins, modify amino acids, generate small peptides, and lower food acidity while simultaneously preventing the growth of other bacteria through antibiosis (Karimi et al. 2005; Sues et al. 2005). Additionally, *M. indicus* can be used to produce various organic acids, such as oxalic acid, malic acid, and succinic acid, which can be used as flavoring agents or preservatives in food (Venkataraman and Vaidyanathan 2023). This species is also used in traditional Chinese medicine to treat various diseases, including leprosy, eczema, psoriasis, ulcers, and abscesses, due to its antifungal properties and ability to heal wounds (Barnharst et al. 2021; Bak 2015). Extracts from *M. indicus* are effective in reducing toothache, impotence, high blood pressure, psoriasis, dermatitis, leprosy, ascariasis, eczema, burns, boils, ulcers, and gynecological infections due to toxin accumulation in the human body’s skin layers, lightening women’s complexion and resuscitating collagen (Satari et al. 2018; ter Borg et al. 1990). It also works effectively for healthy individuals because it provides strong liver support without any side effects. The mitochondrial genome, often referred to as the 'second genome’ of eukaryotes, is a crucial factor in the regulation of growth and development, sustaining homeostasis of the cell and responding to the environment (Murphy 2009; Ernster and Schatz 1981; McBride et al. 2006). It is believed that the mitochondrial genome is an effective tool for studying fungal phylogeny (Li et al. 2022; Li et al. 2022). However, to date, the mitochondrial genome characteristics of early differentiated fungi from the *Mucor* genus are less understood, and only two fungal mitochondrial genomes from the *Mucor* genus have been reported.

## 2. Materials and methods

### 2.1. Sample collection

**Figure 1.**
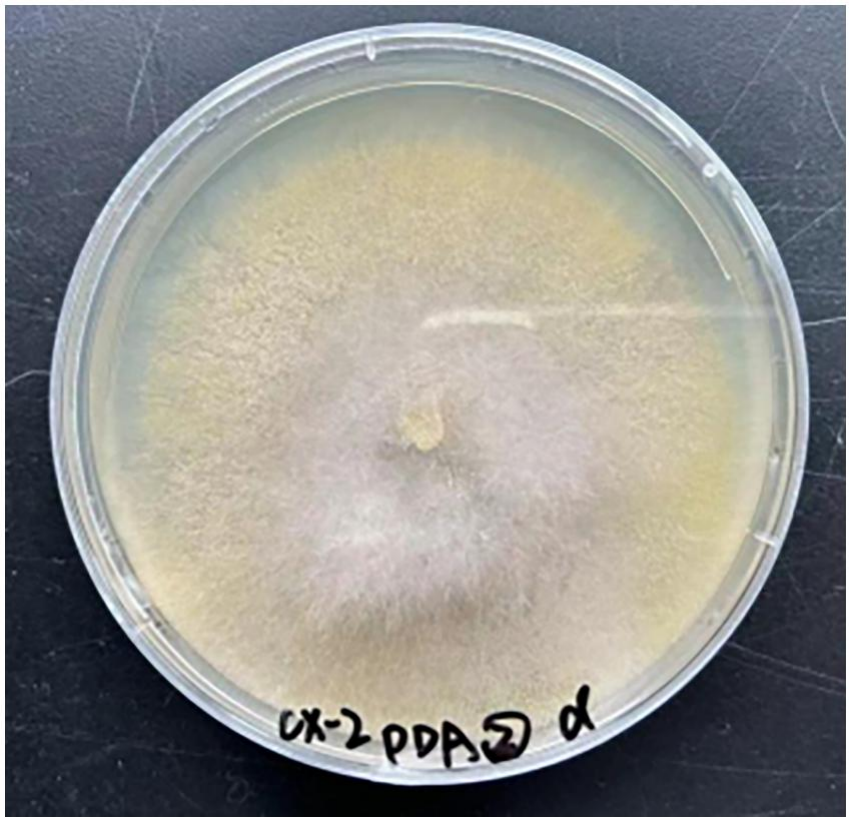
Isolation of *Mucor indicus* mycelia from a wine fermentation system. A photo of the species was taken by Qiang Li.

*M. indicus* was isolated from a wine fermentation system in Luzhou (E 105.40°, N 28.91°), Sichuan, China, in 2023 (Figure 1). The isolated specimens were identified through morphological and molecular evidence (ITS rDNA sequencing). We deposited the specimen at the Culture Collection Center of Chengdu University (contact person: Qiang Li; email: leeq110@126.com) under voucher number Mind1.

### 2.2. Mitochondrial genome assembly and annotation

DNA extraction from *M. indicus* was carried out by using a fungal DNA extraction kit from Omega Bio-Tek (Norcross, GA, USA). For sequencing library preparation, a NEBNext^®^ Ultra^™^ II DNA Library Prep Kit (NEB, Beijing, China) was used in accordance with the manufacturer’s instructions. The Illumina HiSeq 2500 platform (Illumina, San Diego, CA, USA) was utilized for whole-genome sequencing. To ensure data accuracy, ngsShoRT (Chen et al. 2014) was used to filter out low-quality sequences, and AdapterRemoval v2 (Schubert et al. 2016) was used to remove adapter reads. NOVOPlasty v4.3.3 (Dierckxsens et al. 2017) was utilized for de novo assembly of the *M. indicus* mitochondrial genome, employing a k-mer size of 28. The mitochondrial genome was annotated using the MFannot tool (Valach et al. 2014) and MITOS (Bernt et al. 2013), in accordance with our previously described methods (Li et al. 2020; Li et al. 2023). Through the NCBI Open Reading Frame Finder, we were able to predict or modify PCGs or ORFs with a length of more than 100 amino acids (N.R. Coordinators 2017). The functions of PCGs or ORFs were annotated through BLASTP searches against the NCBI nonredundant protein sequence database (Bleasby and Wootton 1990). The exon and intron boundaries of the PCGs were pinpointed using exonerate version 2.2 (Slater and Birney 2005). Using tRNAscan-SE v1.3.1 software, we further predicted and verified tRNA genes in the *M. indicus* mitochondrial genome (Lowe and Chan 2016). A graphical illustration of the mitochondrial genome was generated using OGDraw v1.2 (Lohse et al. 2013). The structures of intron-containing genes were visualized using the PMGmap online web (http://www.1 kmpg.cn/pmgmap, Supplementary Figure S1) (Zhang et al. 2024).

### 2.3. Phylogenetic analysis

The phylogenetic tree was constructed according to previously described methods (Li et al. 2020; Li et al. 2021). The Basidiomycota species Apiotrichum gracile was used as the outgroup. First, individual mitochondrial genes (excluding intron regions), including *atp6*, *atp8*, *atp9*, *cob*, *cox1*, *cox2*, *cox3*, *nad1*, *nad2*, *nad3*, *nad4, nad4L, nad5*, and *nad6* (Katoh et al. 2019), were aligned using MAFFT v7.037 software. Using SequenceMatrix v1.7.8 software, we linked the aligned mitochondrial genes to a single combined mitochondrial dataset (Vaidya et al. 2011). An initial partition homogeneity test was executed to detect any phylogenetic discrepancies between distinct mitochondrial genes. PartitionFinder 2.1.1 was utilized to identify the optimal models of partitioning schemes and evolution for the combined mitochondrial dataset (Lanfear et al. 2017). MrBayes v3.2.6 was utilized to construct phylogenetic trees through the application of Bayesian inference (Ronquist et al. 2012). Two independent runs with four chains (three heated and one cold) each were conducted simultaneously for 2 × 10^6^ generations. Each run was sampled every 100 generations. We assumed that stationarity had been reached when the estimated sample size was greater than 100 and the potential scale reduction factor approached 1.0. The first 25% of the samples were discarded as burn-in, and the remaining trees were used to calculate Bayesian posterior probabilities (BPPs) in a 50% majority rule consensus tree. We also conducted maximal likelihood (ML) analysis to construct a phylogenetic tree using RAxML v 8.0.0 (Stamatakis 2014). Bootstrap values (BS) were assessed through an ultrafast bootstrap approach with 10,000 replicates.

## 3. Results

**Figure 2.**
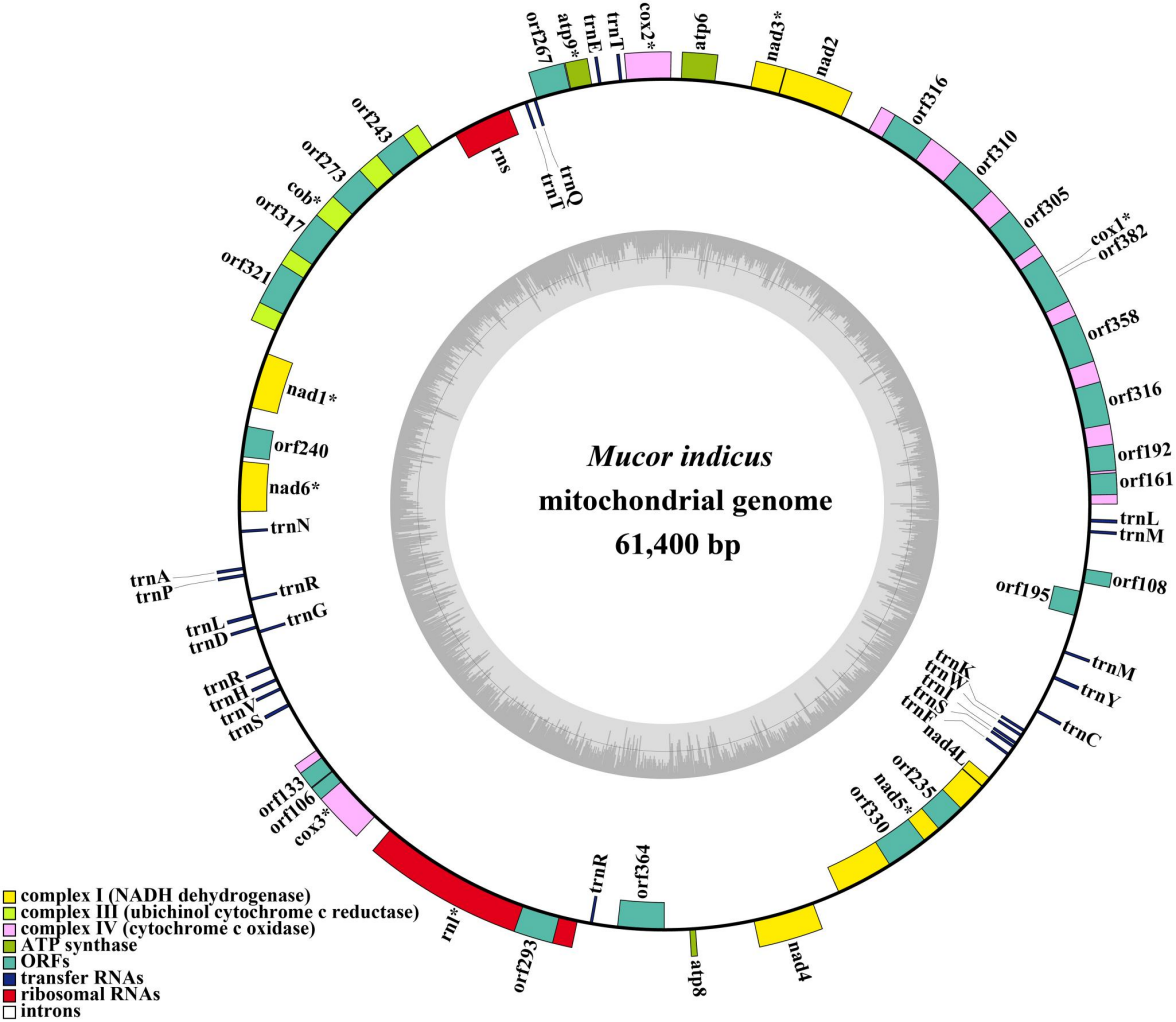
The circular mitochondrial genome map of *Mucor indicus*. Colored blocks outside each ring indicate that the genes are on the direct strand, while colored blocks within the ring indicate that the genes are located on the reverse strand. The inner grayscale bar graph shows the GC content of the mitochondrial sequences. The circle inside the GC content graph marks the 50% threshold.

**Figure 3.**
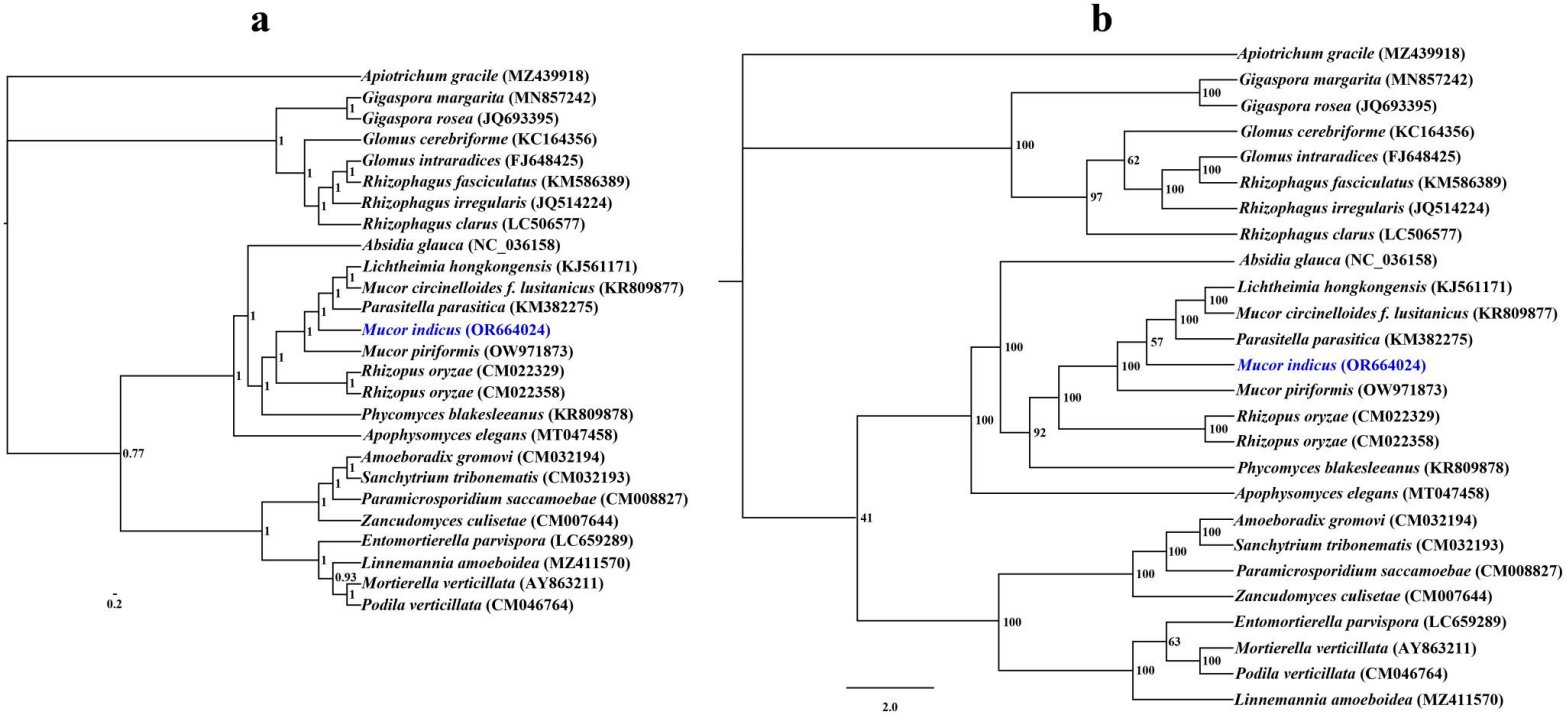
Bayesian inference (a) and maximum likelihood (b) tree generated using 14 concatenated mitochondrial protein-coding genes (*atp6*, *atp8*, *atp9, cob, cox1, cox2, cox3, nad1, nad2, nad3, nad4, nad4L, nad5,* and *nad6*) from *Mucor indicus* and 25 other fungal species. The support values are Bayesian posterior probabilities (BPPs) and bootstrap values (BSs). *Apiotrichum gracile* was used as the outgroup. The accession number information of the sequence is as follows: *Linnemannia amoeboidea* (MZ411570) (Yang et al. 2022), *Glomus cerebriforme* (KC164356) (Beaudet et al. 2013), *Mucor piriformis* (OW971873), *Rhizophagus fasciculatus* (KM586389), *Rhizophagus clarus* (LC506577) (Kobayashi et al. 2018), *Lichtheimia hongkongensis* (KJ561171) (Leung et al. 2014), *Gigaspora rosea* (JQ693395) (Nadimi et al. 2012), *Sanchytrium tribonematis* (CM032193) (Galindo et al. 2021), *Apiotrichum gracile* (MZ439918) (Li et al. 2023), *Mucor indicus* (OR664024), *Glomus intraradices* (FJ648425) (Lee and Young 2009), *Phycomyces blakesleeanus* (KR809878), *Entomortierella parvispora* (LC659289) (Herlambang et al. 2022), *Mortierella verticillata* (AY863211) (Seif et al. 2005), *Podila verticillata* (CM046764) (Morales et al. 2022), *Absidia glauca* (NC_036158) (Ellenberger et al. 2016), *Apophysomyces elegans* (MT047458), *Rhizopus oryzae* (CM022358), *Zancudomyces culisetae* (CM007644) (Nie et al. 2019), *Parasitella parasitica* (KM382275) (Ellenberger et al. 2014), *Paramicrosporidium saccamoebae* (CM008827) (Quandt et al. 2017), *Mucor circinelloides f. lusitanicus* (KR809877), *Amoeboradix gromovi* (CM032194) (Galindo et al. 2021), *Rhizophagus irregularis* (JQ514224) (Formey et al. 2012), *Rhizopus oryzae* (CM022329), and *Gigaspora margarita* (MN857242) (Venice et al. 2020).

A coverage-depth map was generated, and the average depth was 6324.81 × (Supplementary Figure S2). The complete mitochondrial genome was 61,400 bp long, with a GC content of 33%. The total nucleotide composition of the *M. indicus* mitochondrial genome was 33.53% A, 16.61% G, 33.47% T, and 16.39% C. Thirty-six open reading frames (ORFs) were detected in the *M. indicus* mitochondrial genome, which included 14 core PCGs (*cox1*, *cox2*, *cox3*, *atp6*, *atp8*, *atp9*, *cob*, *nad1*, *nad2*, *nad3*, *nad4*, *nad4L*, *nad5*, and *nad6*), 4 free-standing ORFs, and 18 intronic ORFs (Figure 2). These free-standing ORFs mainly encoded proteins with unknown functions. A total of 23 introns were detected in the *M. indicus* mitochondrial genome. Some introns contained one or two intronic ORFs encoding LAGLIDADG-homing endonucleases or GIY-YIG-homing endonucleases. Among the 23 introns detected in the *M. indicus* mitochondrial genome, 19 belonged to Group IB, 2 belonged to Group IA, and 2 belonged to Group ID. Two rRNA genes were detected in the *M. indicus* mitochondrial genome, namely, the small subunit ribosomal RNA (*rns*) and the large subunit ribosomal RNA (*rnl*). In addition, the *M. indicus* mitochondrial genome contained 26 tRNA genes. Both Bayesian inference (BI) and maximum likelihood (ML) methods yielded an identical and well-supported phylogenetic tree, which indicated that *M. indicus* is closely related to *Mucor piriformis* (Figure 3).

## 4. Discussion and conclusion

Through the use of the mitochondrial genome, we can gain a better understanding of the phylogenetic relationships of species (Zhang et al. 2020; Zhang et al. 2019; Zhang et al. 2023; Gao et al. 2024). The lack of a mitochondrial reference genome for Mucoraceae, especially *Mucor* species, restricts the utilization of the mitochondrial genome for classifying and analyzing the phylogenetic relationships of early-diverging fungi. In this study, we newly obtained one complete mitochondrial genome of *Mucor* species. The complete mitochondrial genome was 61,400 bp long, with a GC content of 33%, and contained 14 core protein-coding genes (PCGs), 4 free-standing ORFs, 18 intronic ORFs, 26 tRNAs and 2 rRNA genes. Using the BI and ML phylogenetic inference methods, we were able to generate phylogenetic trees for 25 fungi that differentiated early, with high support rates in major clades; this showed that *M. indicus* is most closely related to *Mucor piriformis*. This research provides useful reference data for the classification and identification of *Mucor* species, thus deepening our knowledge of mitochondrial evolution and the diversity of early-diverging fungi.

## Supplementary Material

Supplemental Material

Supplemental Material

## Data Availability

The genome sequence data that support the findings of this study are openly available in the NCBI GenBank at https://www.ncbi.nlm.nih.gov/under accession no. OR664024. The associated BioProject, SRA, and Bio-Sample numbers are PRJNA1025866, SRR26320847 and SAMN37723662, respectively.
