# Supplementary material for: Characterization of the complete mitochondrial genome of Mucor indicus Lendn. 1930 (Mucorales: Mucoraceae), isolated from the wine fermentation system
Source: Mitochondrial DNA B Resour. 2024 Jun 25;9(6):845–9. doi: 10.1080/23802359.2024.2371376 (PMC11210418; doi:10.1080/23802359.2024.2371376)
Supplement: Supplemental Material [file TMDN_A_2371376_SM2829.docx]

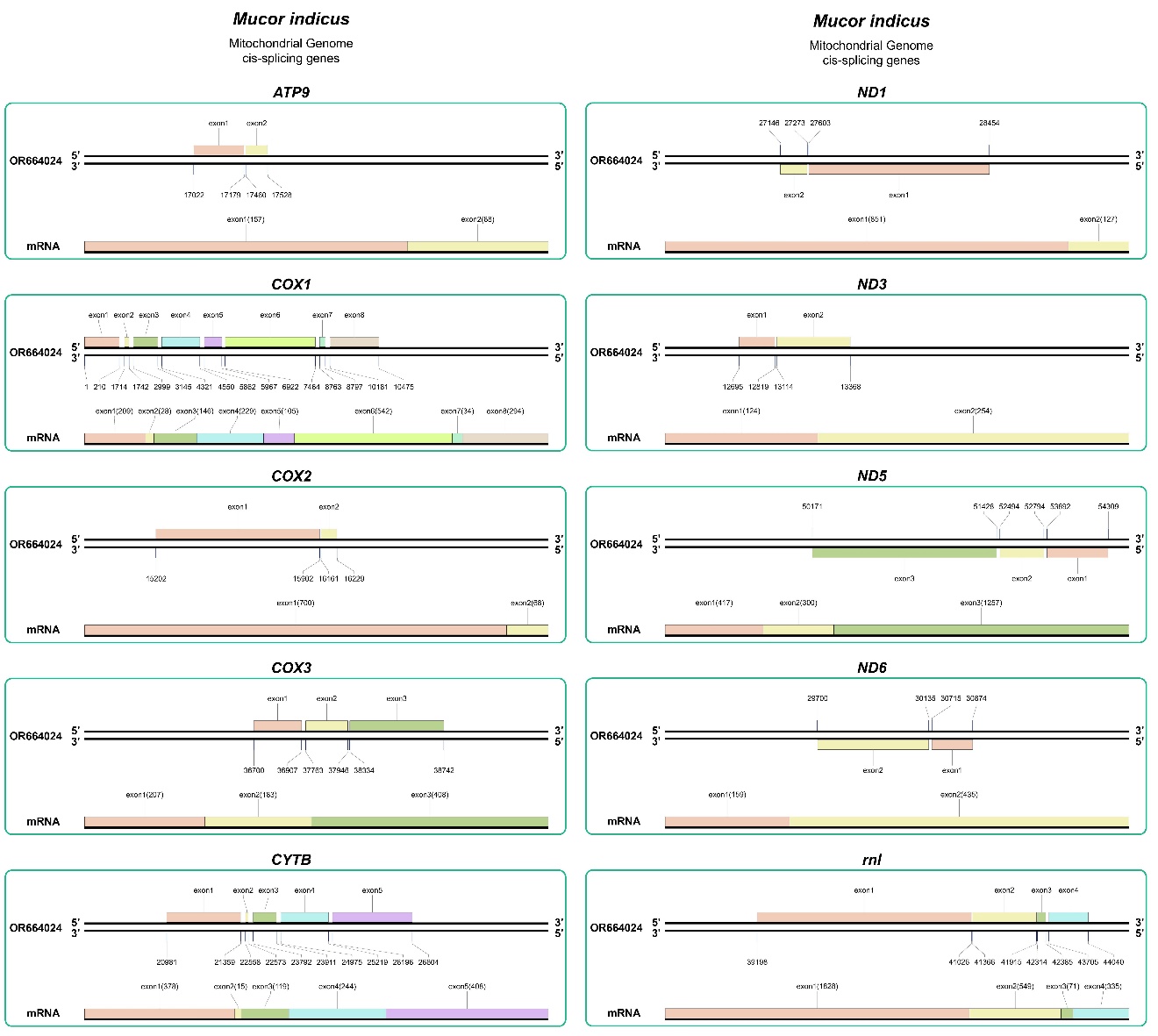


**Supplementary Figure S1** Cis-splicing genes of the *Mucor indicus* mitochondrial genome.


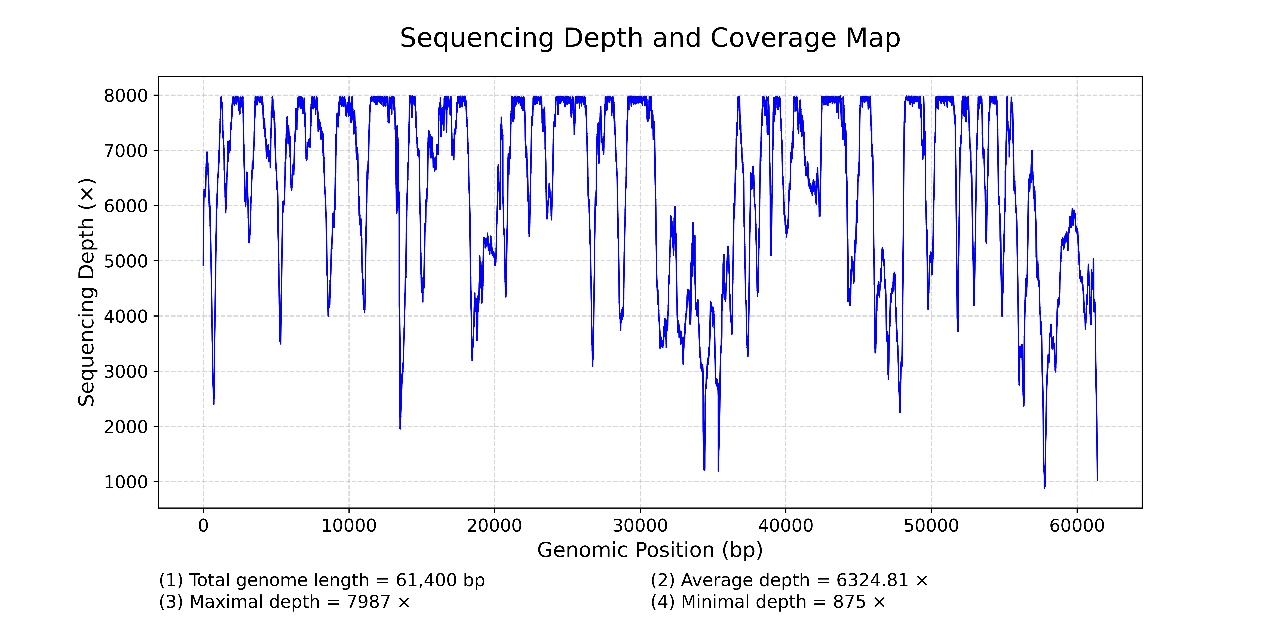


**Supplementary Figure S2** Sequencing depth and coverage map of *Mucor indicus* mitochondrial genome.
