# Supplementary material for: Characterization of the complete mitochondrial genome of Mucor indicus Lendn. 1930 (Mucorales: Mucoraceae), isolated from the wine fermentation system
Source: Mitochondrial DNA B Resour. 2024 Jun 25;9(6):845–9. doi: 10.1080/23802359.2024.2371376 (PMC11210418; doi:10.1080/23802359.2024.2371376)
Supplement: Supplemental Material [file TMDN_A_2371376_SM2825.pdf]

CERTIFICATE

OF ENGLISH LANGUAGE EDITING

Characterization of the complete mitochondrial genome of Mucor indicus Lendn. 1930 (Mucorales: Mucoraceae), isolated from the wine fermentation system

Mucor indicus Lendn. 1930 has been widely used in food fermentation; however, its mitochondrial genome characteristics are not well understood. In this study, the complete mitochondrial genome of M. indicus was obtained, which was 61,400 bp in length with a GC content of 33%. The M. indicus mitochondrial genome was found to contain 14 core protein-coding genes, four free-standing ORFs, 18 intronic ORFs, 26 tRNAs, and two rRNA genes. Phylogenetic trees were generated for 25 early-differentiated fungi using the Bayesian inference (BI) method, which demonstrated that M. indicus is closely related to Mucor piriformis. This study provides useful information for the classification and evolution of Mucor species or other early-differentiated fungi.

This document certifies that the manuscript listed above was copy edited for English language by LetPub, with regard to grammar, punctuation, spelling, and clarity. Documents receiving this certification should be regarded as having undergone professional editorial revision for English language before submission. However, the authors may accept or reject LetPub′ s suggestions and changes at their own discretion and LetPub does not have editorial control over the submitted documents. Submitted documents may have new text that was not provided to LetPub for review. Please use the verification link below to determine the validity of the submitted version.

June 7, 2024

Date of Revision

LetPub

LetPub is an author service brand owned and operated by Accdon LLC.
Tel: 1-781-202-9968 Email: info@accdon.com
Address: 400 Fifth Ave, Suite 530, Waltham, MA 02451, United States

This manuscript has been individually edited for grammar, punctuation, spelling, and clarity.

© 2024 Accdon, LLC.
